# Pressure–temperature evolution of primordial solar system solids during impact-induced compaction

**DOI:** 10.1038/ncomms6451

**Published:** 2014-12-03

**Authors:** P. A. Bland, G. S. Collins, T. M. Davison, N. M. Abreu, F. J. Ciesla, A. R. Muxworthy, J. Moore

**Affiliations:** 1 Department of Applied Geology, Curtin University, GPO Box U1987, Perth, Western Australia 6845, Australia; 2 Impacts & Astromaterials Research Centre (IARC), Department of Earth Science & Engineering, Imperial College London, South Kensington Campus, London SW7 2AZ, UK; 3 Earth Science Program, Pennsylvania State University—Du Bois Campus, Du Bois, Pennsylvania 15801, USA; 4 Department of Geophysical Science, University of Chicago, 5734 South Ellis Avenue, Chicago, Illinois 60430, USA

## Abstract

Prior to becoming chondritic meteorites, primordial solids were a poorly consolidated mix of mm-scale igneous inclusions (chondrules) and high-porosity sub-μm dust (matrix). We used high-resolution numerical simulations to track the effect of impact-induced compaction on these materials. Here we show that impact velocities as low as 1.5 km s^−1^ were capable of heating the matrix to >1,000 K, with pressure–temperature varying by >10 GPa and >1,000 K over ~100 μm. Chondrules were unaffected, acting as heat-sinks: matrix temperature excursions were brief. As impact-induced compaction was a primary and ubiquitous process, our new understanding of its effects requires that key aspects of the chondrite record be re-evaluated: palaeomagnetism, petrography and variability in shock level across meteorite groups. Our data suggest a lithification mechanism for meteorites, and provide a ‘speed limit’ constraint on major compressive impacts that is inconsistent with recent models of solar system orbital architecture that require an early, rapid phase of main-belt collisional evolution.

## Abstract (web summary)

Collisions between primordial planetesimals led to the formation of our asteroids and meteorites. Here, the authors use modelling to explore the compaction of planetsimals, tracking how pressure, temperature and porosity may have varied during the impacts, helping interpret early Solar System processes.

## Graphical abstract

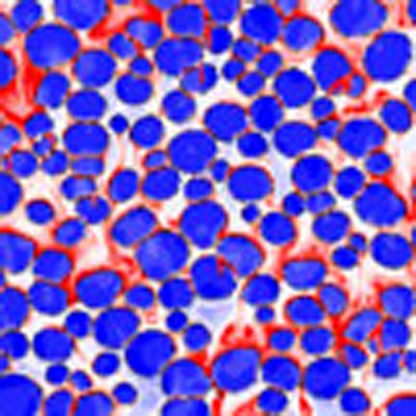

Chondritic asteroids are among the most primitive objects in the solar system, containing a unique record of solar system formation. Three processes dominated their evolution as geological bodies—thermal metamorphism, aqueous alteration and impacts. Meteorite classification schemes are based on the degree to which a given sample was exposed to these processes. The impact record in chondrites has been studied in detail^1^. Shock metamorphism is not considered a dominant factor in the evolution of the most primitive meteorites, the carbonaceous chondrites (CC), as 85% are ranked S1 (‘unshocked’; <4–5 GPa) or S2 (‘very weakly shocked’; 5–10 GPa)^1^,^2^. Associated low-intensity collisions are thought to leave a chondrite essentially unscathed, with no effects from local pressure–temperature (PT) excursions, and minimal post-shock thermal metamorphism^1^. Primitive (that is, unequilibrated) ordinary chondrites rank at higher shock levels: 50% are S3 (‘weakly shocked’; 10–20 GPa)^1^. Shock level is calibrated against shock recovery experiments on non-porous or low-porosity crystals and rocks^1^, but porous objects respond very differently to impact than non-porous objects^3^,^4^. When shocking a porous material, extra *P*d*V* work is expended to crush out the pore space. After release from the shocked state, that extra work (termed the ‘waste heat’) heats the material, thus porous material will reach higher temperatures than a non-porous material in a similar impact. Although they are now lithified (often low porosity) rocks, chondrite precursors were highly porous^5^,^6^,^7^,^8^.

In addition, chondrites are bimodal materials: zero-porosity mm-sized spherical chondrules set in an aggregate composed of sub-μm monomers. But the level of impact processing in chondrites is determined by shock metamorphic textures in large (>50–100 μm) grains (for example, chondrule olivines), accessible via optical microsocpy^1^,^2^. The assumption has been that if chondrules are unshocked, so is matrix. The possibility that the shock record might be different in matrix has not been considered, and with notable exceptions^9^,^10^,^11^, the extent of shock metamorphism in matrix has not been explored.

Alongside petrographic observations and experimental impact studies, computational modelling has been utilized extensively to understand impact shock, generally applied to non-porous targets, but most recently in porous planetesimals^11^. In these models, porosity is parameterized, and derived PT estimates are ‘bulk’ values, averaged over large (asteroidal) scales, making it difficult to translate model predictions to meteorite observations. No numerical studies have attempted to resolve shock in the bimodal materials that were chondritic precursors. This is unfortunate given that they were arguably the starting point for all inner solar system objects.

An obstacle to understanding impact effects in porous meteorites has been the absence of direct information on the nature of the porosity prior to the shock event^3^. But a methodology that quantitatively relates rock fabric intensity to net compression^7^ has overcome this obstacle, allowing us to reconstruct a pre-compaction porosity for matrix and for the parent body as a whole. As outlined in that work^7^, it is possible to reconstruct a pre-compaction matrix porosity by deriving the ratio of final (that is, post-compaction) lengths to initial (pre-compaction) lengths of notional lines perpendicular to the matrix uniaxial fabric. This ratio (*Z*) is a quantitative measure of the degree of compaction. Given the current observed (final) abundance of matrix (*A*_mf_), and current (final) bulk porosity (*φ*_bf_), we can estimate current (final) matrix porosity (*φ*_mf_) (assuming chondrule porosity ~0%). Given *Z* and *φ*_mf_, the initial (pre-compaction) matrix porosity (*φ*_mi_) is given by *φ*_mf_*Z*+(1−*Z*). It follows that initial bulk porosity (*φ*_bi_) is given by


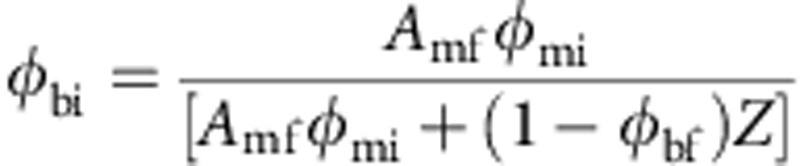


and initial matrix abundance (*A*_mi_) by *φ*_bi_/*φ*_mi_.

Thus far the early evolution of the material properties of solar system solids has been explored largely via experiments with analogue materials^5^. Most recently, impact compaction experiments compared final bulk porosities with chondrite values^8^. The saturation limit of the porosity varies for different materials: 0.68–0.42 at a pressure of 1 MPa^8^. The upper bound appears to be a closer approximation to *φ*_mi_ for chondrite parent bodies. In OCs, *φ*_bf_ is typically ~5% (ref. 12), but there are outliers with values up to 21% (ref. 13). For the most porous OCs it has been shown that this is primordial (not fracture) porosity^13^. Similarly, for anhydrous CCs there is a large range: *φ*_bf_=7–28% for CO chondrites, and 4–28% for CV chondrites^14^. Using the relation outlined above, assuming (conservatively), no compaction (*Z*=1, and *φ*_mf_*=φ*_mi_), it is apparent that current matrix porosity (*φ*_mf_) in the most porous OCs must be 60–70%; 60% in the most porous CVs and 66–70% in the most porous COs. In the CV chondrite Allende, where a detailed rock fabric analysis was performed^7^,^15^, and consistent matrix fabrics observed across the sample, degree of compaction consistent with *Z*=~0.5 was defined^7^ (that is, compaction had roughly halved the volume of the matrix aggregate). A *φ*_mi_ value of 70–80% was derived^5^. The data from compaction experiments^8^, fabric analysis^7^ and an overview of literature porosity, appear consistent with *φ*_mi_=~70% across most chondrite groups.

If chondrite precursors were initially highly porous, how were they compacted to the (generally) low-porosity rocks that we see today? Recent studies^8^ favour impacts as a compaction mechanism, and several other observations support that conclusion. Lithostatic pressure (gravitational compression) was trivial (~1 MPa at the centre of a 100-km radius asteroid, equivalent to a depth of 10 m on Earth^16^), indicating that it was not a significant factor in chondrite compaction. In addition, gravitational compaction would generate parent bodies with meteorites from the interior being more compacted (and showing more thermal metamorphism), than meteorites sourced from nearer the surface of the object. This is not observed. There is a lack of positive correlation between compaction and thermal metamorphism in OCs^12^, COs^14^ or CVs^14^. In the CVs there is a negative correlation: unmetamorphosed CV_r_s are highly compacted (3.6% porosity), while more metamorphosed CV_o_s are not (averaging 19.7%)^14^. Taken together, these observations are consistent with impact-induced compaction of initial high-porosity material occurring on large (parent body?) scales.

Here we use numerical modelling to study the effects of impact-induced compaction on primordial chondritic materials. Our goal was to achieve sufficient resolution in simulations to allow a comparison with meteorite petrography observed in a thin section (100 μm). Although low velocity impacts are expected to produce minor effects in CCs^1^,^2^, resolving at this finer ‘mesoscale’, we find that this is not the case. Even at 1.5 km s^−1^, porous matrix can experience peak *T*>1,000 K as it compacts. PT varies by >10 GPa and >1,000 K over ~100 μm. Chondrules act as heat-sinks, so temperature excursions are brief. These results have implications for key aspects of the chondrite record, and provide a ‘speed limit’ constraint on the initial collisional evolution of planetesimals.

## Results

### Mesoscale simulations

We have developed a two-dimensional (2D) ‘mesoscale’ numerical approach that allows quantification of peak shock pressures (*P*(peak)) and post-shock temperatures (immediately after passage of the shock wave) (*T*(final)) across a computational mesh composed of a bimodal mixture of chondrules surrounded by a highly porous matrix using the iSALE hydrocode^17^,^18^,^19^. Our approach complements and extends recent experimental work that tracked porosity in analogue materials^8^, allowing us to observe how pressure, temperature and porosity vary during an impact within and between chondritic components. Tracer particles recorded the peak- and post-shock state of the matrix and chondrule material, from which the bulk state was determined. We conducted 16 simulations (Table 1) that spanned a range in impact velocity (0.75–3 km s^−1^) and initial matrix volume fraction (70 and 33%) to simulate impacts that generated post-compaction matrix volume fractions consistent with CC-like objects and ordinary chondrite (OC)-like objects, respectively. An ANEOS-derived equation of state table for forsterite^20^ was used to describe the thermodynamic response of the non-porous disks and the solid component of the matrix. We also conducted five simulations with serpentine matrix and forsterite chondrules (to approximate CM and CR chondrites), using ANEOS input parameters for serpentine^21^. Impact heating will lead to dehydration of hydrous phases, releasing water that may cause textural modifications, but computational limitations meant that we could not model this process explicitly. Compaction of porosity and material strength were modelled using methods described in earlier work^18^,^19^. We take *φ*_mi_=70% as a standard value in our simulations, with selected comparison runs at *φ*_mi_=60% and *φ*_mi_=80%. Initial temperature was 300 K (an argument can be made that this should be higher, given that peak metamorphic temperature in CV chondrites exceeded 600 K, and compaction post-dated formation of secondary minerals). We explored the effect of varying *T*(initial). For small (100 K) variations, temperature changes predicted by the models were independent of the starting temperature, for example, 100 K lower *T*(initial) would reduce final temperatures by that amount.

**Figure 1:**
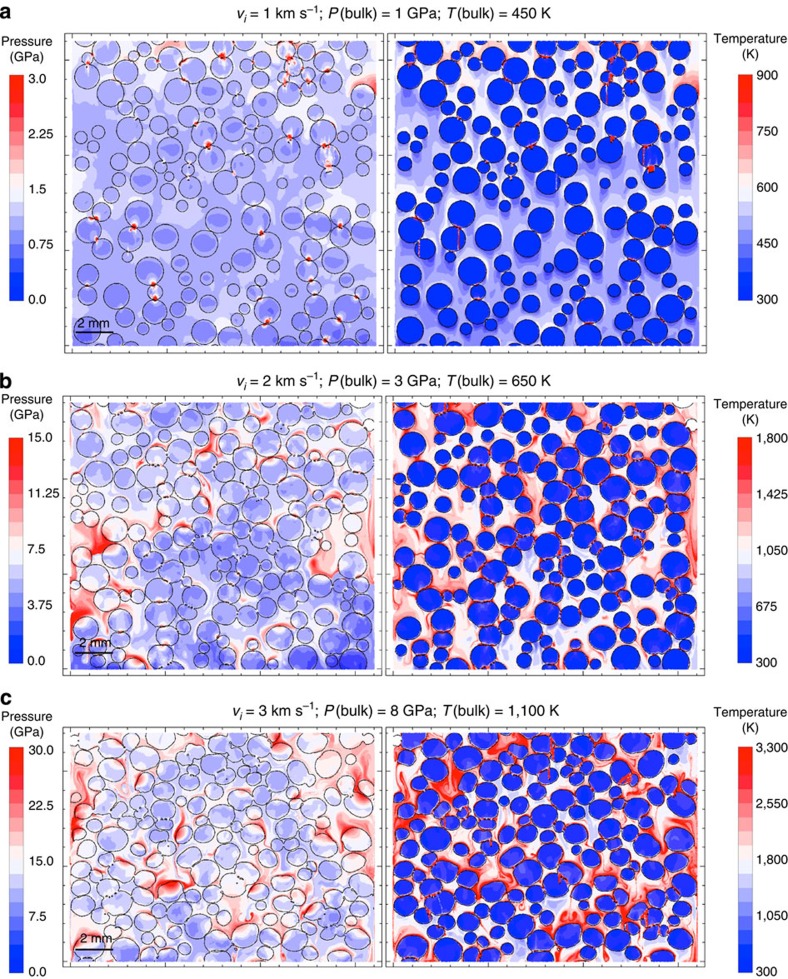
Variation in peak-shock pressure and peak temperature in mesoscale simulations. In the examples shown, matrix is composed of forsterite, initial matrix volume fraction is 70%, initial matrix porosity is 70% and starting temperature is 300 K. (**a**) 1 km s^−1^ impact results in bulk shock pressure ~1 GPa; (**b**) 2 km s^−1^ impact results in *P*(bulk) ~3 GPa; (**c**) 3 km s^−1^ impact results in *P*(bulk) ~7 GPa. The images illustrate the variation in *P*(peak) and *T*(peak) recorded by a representative portion of the sample after passage of the shock wave. Note the change in pressure and temperature scale between frames. The extreme variability in matrix PT over short lengthscales is apparent, indicated by swirling contours in matrix. Our simulations reproduce a variety of chondrule/matrix textures observed in the chondrite record (elongate or oriented chondrules; matrix ‘flowing’ between chondrules; indented chondrules). Chondrule indentation textures become apparent at 2 km s^−1^ (1.5 km s^−1^ at lower matrix vol% (for example, OCs)); internal chondrule *P*(peak) ~5 GPa.

Our simulations allow us to observe how pressure, temperature and porosity vary during an impact within and between chondritic components, observing detail down to the 100-μm level (Fig. 1). Although low velocity impacts are expected to produce minor effects in CCs^1^,^2^, resolving at this finer ‘mesoscale’, we find that this is not the case. Predictably, matrix porosity is removed rapidly, decreasing from 40–50% at 0.75 km s^−1^ to ~15% at 1.5 km s^−1^. But matrix and chondrules experience very different conditions, particularly with respect to temperature, in all scenarios (Fig. 1). A 2.0-km s^−1^ event into a 70% forsterite matrix precursor (Fig. 1b) with *φ*_mi_=70% generated chondrule *P*(peak) of 6.5±1.8 GPa: barely detectable in a typical petrographic study^1^,^2^. However, matrix experiences *T*(final) of 1,100 K while chondrules are virtually unheated (Δ*T*(final) ~70 K). We also observe significant heterogeneity in PT conditions over short (100 μm) lengthscales. Taking the same example (2.0 km s^−1^ and 70% initial matrix volume fraction: Fig. 1b), we observe a 7–18-GPa range of *P*(peak) over 100 μm in matrix (average matrix *P*(peak) is 8.4±2.6 GPa in this simulation), and a 700–2,000-K variation in matrix *T*(final) over similar lengthscales (average matrix *T*(final) is 1,100 K). Chondrule deformation (and flattening) is apparent at velocities of 2 km s^−1^ and higher. The assumed Hugoniot Elastic Limit for the chondrules (~5 GPa) is exceeded in these higher-velocity simulations, that is, the pressure is so high that they are deformed in a plastic, rather than in a brittle manner, despite the high strain rate. We also note that chondrule edges experience higher shock than chondrule interiors. This occurs where chondrules impact and indent one another (leading to local *P*(peak) spikes up to ~12 GPa in the 2-km s^−1^ simulation), but it is also a general feature, as matrix compacts around chondrules. Although average chondrule *P*(peak) is 6.5 GPa at 2 km s^−1^, interiors only see 4–5 GPa, while chondrule edges range up to 8 GPa (it is probable that the real value is higher, but spatial resolution is limited by computational costs). Finally, with regard to the serpentine matrix scenarios, at low shock pressure there is little difference in matrix *T*(final) between serpentine and forsterite. In simulations ≥2 km s^−1^ temperature is buffered by phase changes in serpentine, reducing matrix temperatures in these higher-shock scenarios. However, even here the same general features remain: high matrix heating alongside ‘cold’ chondrules, and significant PT heterogeneity over short lengthscales.

Previous studies examined shock effects in porous targets, or non-porous targets, but not mixtures. It is known that impacts into porous materials result in much more heating than impacts into non-porous materials, as shock energy is expended in collapsing pores^3^,^4^. Impacts into a uniformly porous asteroid will compact that material, heating a large volume around the crater that then cools and equilibrates over timescales up to 10–100 Myr^11^. Primitive chondrites are compacted, but are texturally and compositionally unequilibrated. Our data indicate that impact-induced compaction in chondrites occurred without generating macroscopic thermal metamorphic textures, or chemical equilibration, because of the juxtaposition of two materials with vastly different initial porosities and bulk properties. In the chondrite scenario, chondrules act as a heat sink. Matrix is heated rapidly to 100's of degrees above ambient, but because of the proximity to ‘cold’ chondrules, it cools rapidly. Rather than slow cooling of a large volume^11^, we see sub-mm heterogeneities that equilibrate in <10 s. So unless bulk *T*(final) exceeds temperatures typical of CC peak metamorphism (a condition we observe only in scenarios with impact velocity >2–2.5 km s^−1^), macroscopic effects will not be apparent.

### Electron microscopy

**Figure 2:**
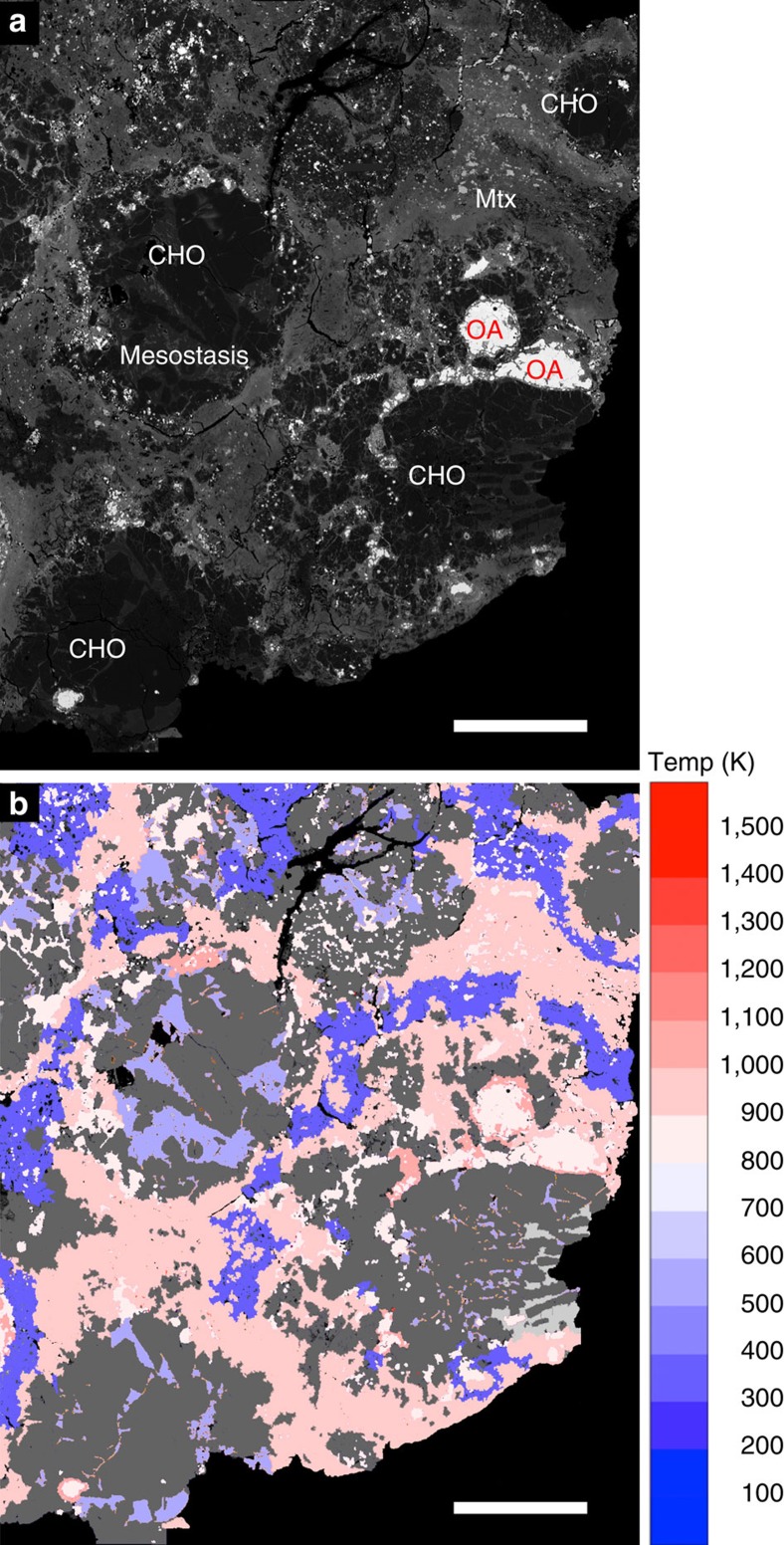
Back-scattered electron (BSE) and temperature images of CR2 chondrite GRA 06100. (**a**) BSE image showing chondrules (CHO), mesostasis, opaque assemblages (OA) and matrix (Mtx). (**b**) Temperature map of the same area. Scale bar, 0.5 mm. Chondrule olivines (gray-scale regions) show no evidence of heating or shock. Temperatures were estimated based on the crystallographic transitions of aliettite (423, 493, 878 and 1,178 K)^24^; presence of near-end member ankerite (*T*>1,100 K)^25^; thermal decomposition of pentlandite (*T*>873 K)^27^; scorzalite–lazulite solid solution (*T*>758 K)^26^.

Although the effect of shock on fine-grained crystalline solids has been investigated^22^, there are few detailed transmission electron microscopy (TEM) studies of shock metamorphism in the meteorite matrix^9^. And while petrographic studies of experimentally shocked chondrites are informative^23^, the ‘unshocked’ starting point in this work is a meteorite that has already experienced significant compaction. Given the limited literature, we conducted our own coordinated field emission (FE)-electron probe microanalysis (EPMA), FE-scanning electron microscope (SEM) and focused ion beam (FIB)/TEM study of a CR2 (GRA 06100), focussing specifically on how impact-induced processes may have affected sub-μm materials in this rock. We devised a novel methodology that used temperature-related crystallographic transitions to quantify matrix temperatures at fine scale. To select regions to extract FIB sections, we first built a map based on FE-SEM back-scattered electron and energy-dispersive spectrometry (EDS). The results of the FIB analysis guided us in interpreting detailed mineralogy from the original map. This combined approach enabled us to draw the first detailed isotherm map for a chondrite, in this case GRA 06100 (Fig. 2) (a follow-up study^10^ in which an additional eight FIB sections were extracted found that the mineralogy was consistent with expectations at all the new sites).

No evidence of heating was found in chondrule olivine and pyroxenes. GRA 06100 matrix consists of sub-micron, diffuse masses that have compositions ranging from low-total, Mg, Al-bearing, saponite to ferroan serpentine under a 10-μm EPMA beam. The porosity and mineralogy of GRA 06100 matrix is variable, containing deformed SiO_2_ (grains contain nanocrystalline and amorphous regions), Fe-silicide, nanophase fayalitic olivine and ferrosilite, non-stoichiometric ferromagnesiosilica grains, as well as amorphous silicates and phyllosilicates with variable amounts of Al. Although Fe-rich serpentine is the most common matrix phyllosilicate, the smectite aliettite was identified in several regions of the matrix, using a combination of TEM/EDS and crystallography. Aliettite undergoes four endothermic transitions (150, 220, 605 and 905 °C)^24^, which are of use in determining the minimum temperature reached in a region of matrix. Aliettite recording 220 °C was found in close proximity to aliettite recording 605 and 905 °C.

Near-end member ankerite was found at the edge of a large metal nodule, located <20 μm from unheated chondrule olivines. Ankerite of this composition is predicted to occur at *T*>827 °C (ref. 25). Also <20 μm from unheated chondrule olivines, the high-temperature silicate-sulfate–phosphate scorzalite–lazulite was found. The observed compositions in the scorzalite–lazulite solid solution correspond to *T*>485 °C (ref. 26). Finally, pentlandite, the most common sulphide in CR chondrites, is absent from GRA 06100. Where sulphides are present, they consist of pyrrhotite and partly oxidized Fe–sulfides sub-domains. Thermal decomposition of pentlandite occurring in opaque assemblages in GRA 06100 occurs at *T*>600 °C (ref. 27).

On the basis of the distribution of different minerals of selected areas of the thin section, we produced a thermal contour map (Fig. 2b). We estimate that ~46% of the section shows no evidence of heating, ~12% is consistent with 493 K≤*T*≤873 K, ~28% 873 K≤*T*≤1,023 K, ~9% 1,023 K≤*T*≤1,178 K and ~5% records temperatures ≥1,178 K. However, the great diversity of mineral assemblages identified in matrix indicates that more FIB sections are needed to fully constrain the temperature regimes recorded by fine-grained material. Note: although phase transition temperatures are defined at low pressure, the comparison with model results is valid as we are comparing to post-shock/pre-equilibration temperatures (that is, low pressure after shock passage but before thermal equilibration between chondrules and matrix).

### Macroscale simulations

**Figure 3:**
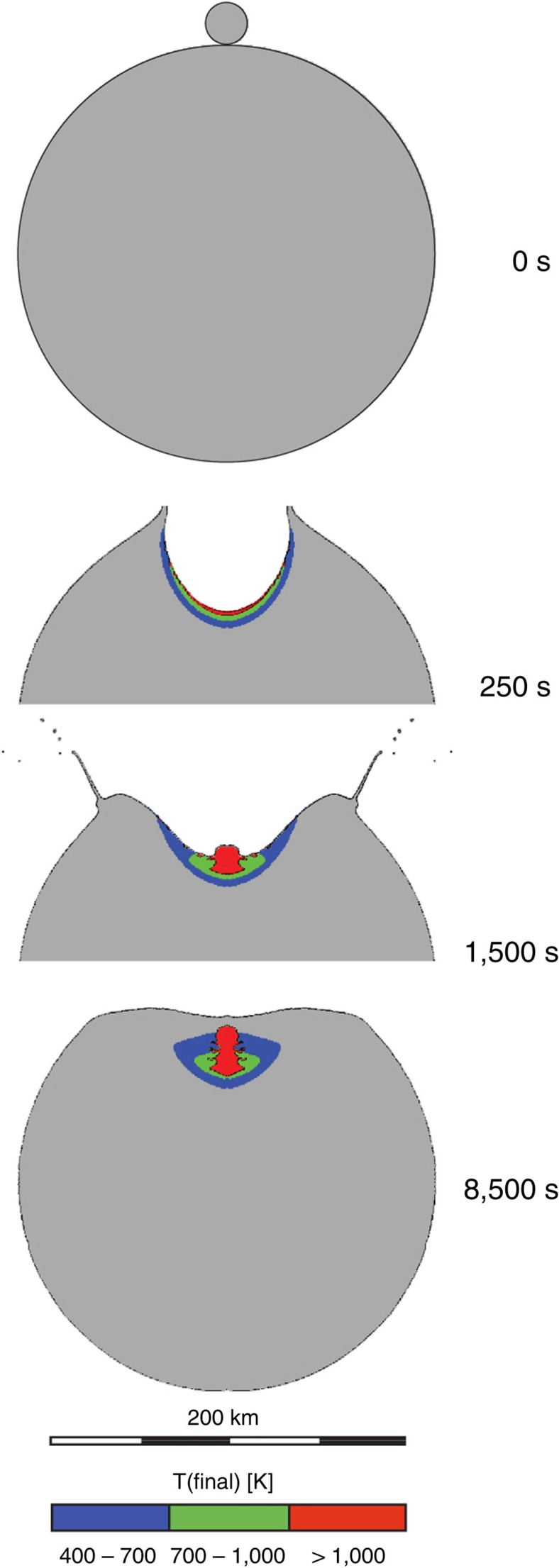
Translating mesoscale observations into asteroid-scale impacts. Macroscale hydrocode modelling of impacts between porous planetsimals. In an impact on a real planetesimal, the shock wave attenuates rapidly with distance, but the peak shock PT conditions are similar to the planar impact scenario. Macroscale models consider impact velocities from 1 to 10 km s^−1^ for both OC-like (20% initial bulk porosity) and CC-like (50% initial bulk porosity) objects; assume vertical impact; and with target planetesimals of 200–500 km diameter, and impacting planetesimals of 10–150 km in diameter. This example involves CC-like objects impacting at 4 km s^−1^. The blue region logs tracers at 400 K<bulk *T*(final)<700 K: the range consistent with meteorite data. Of the material that is compacted to meteorite-like porosities, we assume (conservatively) that if >20% experienced bulk *T*(final) >700 K (shown here in green (700–1,000 K) and red (>1,000 K)) it would be observed in the meteorite record. This corresponds to velocities exceeding 3 km s^−1^ in modelled impacts (Fig. 4). Similar velocities are derived from OC-like simulations.

**Figure 4:**
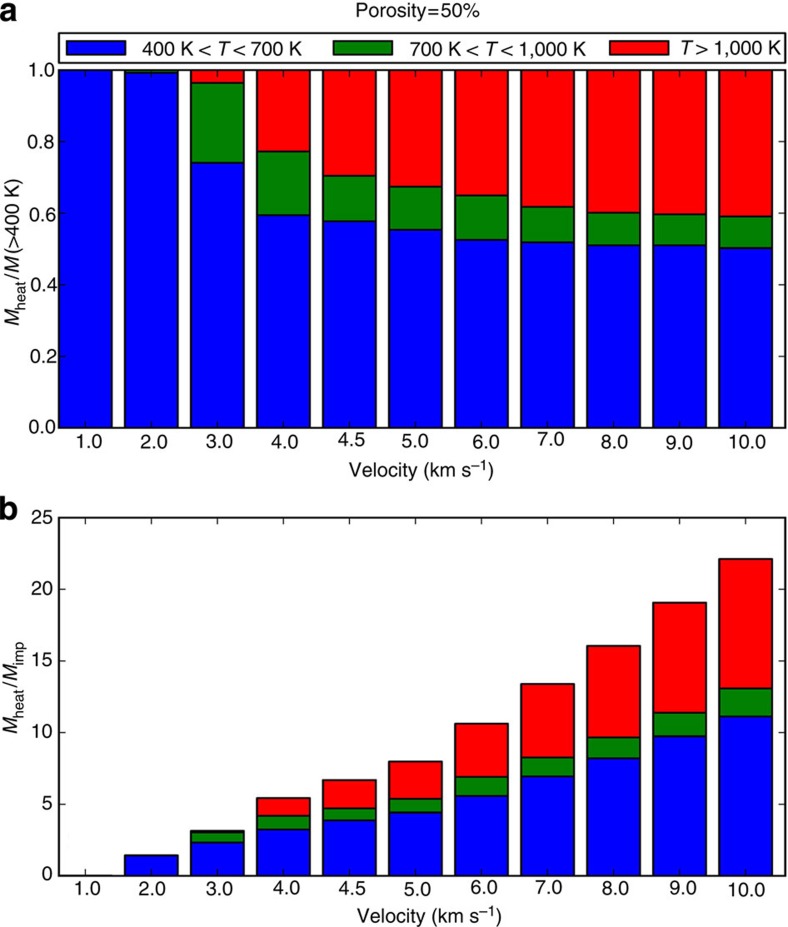
Fraction of material heated to varying temperature in macroscale collisions. Results are shown for head-on collisions between two planetesimals with mass ratios of 1:1,000 and with initial porosities of 50% (CC-like simulation). Equivalent simulations were performed for OC-like (20% initial bulk porosity) objects, and for a range of other target and impactor diameters. Results are normalized by both the impactor mass (**b**) and the mass of material heated by 100 K, which corresponds to a bulk porosity reduction of ~10% (**a**). The fraction of material heated above 700 K exceeds 0.2 at a vertical impact velocity of ~3 km s^−1^. Similar velocities are derived from OC-like simulations. The 2D axial symmetry of the numerical model enforces head-on collisions between planetesimals in the macroscale simulations. In reality, the average impact angle is 45° to the target plane. The volume of material heated to a given temperature also scales with a vertical component of impact velocity in the same way as for vertical impacts^28^. Thus, a 3-km s^−1^ vertical impact is approximately equivalent to a 45° impact at 3/sin(45)=4.25 km s^−1^.

In addition to the mesoscale simulations, a suite of ‘macroscale’ planetesimal collision simulations were performed (for example, Fig. 3). Tracer particles were used to track planetesimal material exposed to different bulk post-shock temperatures for comparison with the mesoscale simulation results and meteoritic constraints (Fig. 3). The mass of material in the range 400 K<bulk *T*(final)<700 K is shown in blue; material that experienced bulk *T*(final) >700 K is shown in green (700–1,000 K) and red (>1,000 K). Figure 4 shows the total mass of material heated to different final bulk temperatures from an example suite of collisions (1:1,000 impactor-to-target mass ratio; 50% initial porosity in impactor and target) at various impact speeds using the same colour coding. All macroscale simulations assumed head-on collisions between planetesimals, enforced by the 2D axial symmetry of the numerical model. In reality, the most common impact angle is 45° to the target plane. Numerical simulations of oblique impacts on planar surfaces suggest that the volume of material heated to a given temperature scales with vertical component of impact velocity in the same way as for vertical impacts^28^. Hence, in the context of Fig. 4, a 3-km s^−1^ vertical impact is approximately equivalent to a 45° impact at 3/sin(45)=4.25 km s^−1^.

## Discussion

Our observations have a number of implications for understanding the chondrite record: lithification of primordial materials may have been a natural outcome of transient heating of matrices, driven by collisional compaction. Hydrothermal alteration has been suggested as a mechanism for meteorite lithification, but some primitive meteorites have escaped this process and yet are consolidated rocks. Lithostatic pressure was minimal^16^, and towards the surface—where primitive chondrites are expected to originate—it would have been even lower. Yet chondrite lithification was efficient even at fine scales: matrices are lithified. Although collisional compression has been proposed as a mechanism^16^, impact shock has generally been excluded as few CCs show macroscopic shock indicators. But our data indicate that this is not a valid constraint when considering the response of matrix to shock. In addition, experiments examining the sintering behaviour of olivine have found that it is favoured where a fine-grained (micron-scale) aggregate is rapidly heated to high *T*^29^: a situation identical to that encountered by high-porosity matrix during impact-induced compaction.

Our results have implications for chondrite palaeomagnetism. Magnetic fields may be recorded at the grain scale without raising the bulk temperature above the Curie point; and this can occur without generating obvious macroscopic shock metamorphic textures (that is, it may have affected meteorites that have been classified as low shock). It is known that an ambient magnetic field at the time of impact may be amplified or even produced by the impact itself^30^. Large impact-generated fields are possible^31^. But impact-generated fields have not been considered a significant factor in the palaeomagnetic record of primitive chondrites for two principal reasons. It was thought that shock pressures >40 GPa^30^ were required to generate temperatures high enough to impart a thermoremanent magnetization (TRM). In these highly energetic events post-impact cooling times would be long (perhaps 10^5^–10^7^Myr). But impact-generated fields are brief (<mins). An additional reason is the absence of macroscopic shock textures in most CCs^30^. Our work indicates that these arguments should be re-examined. We show that it is necessary to model PT evolution at the meteorite scale, in order to constrain laboratory measurements at the meteorite scale. The absence of shock textures in chondrules does not inform our understanding of matrix PT history. The bulk rock will respond as expected in the literature, but on a finer scale, matrix will experience rapid heating and cooling, prior to equilibrating to bulk *T*(final) in <10's of seconds. If the principal magnetic carrier phase was hosted in matrix (as is often the case), then the palaeomagnetic record may well have been modified during compaction. Equally, a matrix phase could record an impact-generated field with a duration of 10's of seconds to minutes. A rock could acquire a TRM during rapid cooling of matrix following impact-induced compaction.

**Figure 5:**
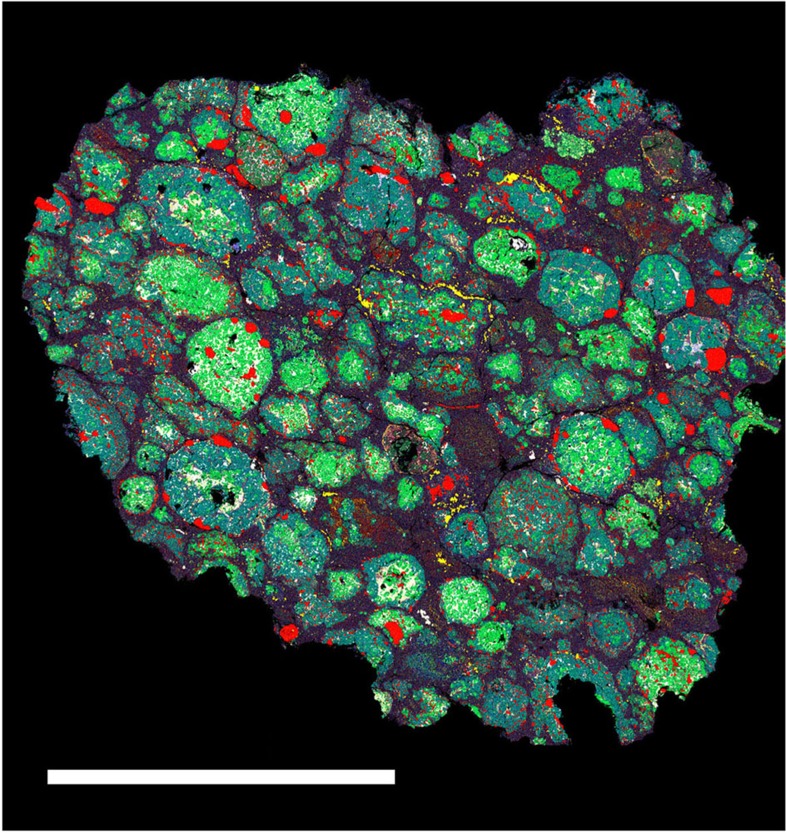
Chondrule textures in the CR2 chondrite Renazzo. Flattened and indented chondrules highlighted in an element map of Renazzo (green:Mgl yellow:Ca; white:Al; red:Fe; blue:Si); textures similar to the 2-km s^−1^ simulation shown in Fig. 1b. Scale bar 6 mm.

Model results showing PT heterogeneity at fine scale and high transient matrix temperatures suggest a unified explanation for anomalous features of chondrite petrography and texture that previously have required complex mechanisms (involving accretion, disruption and dispersal, heating or shock processing in the nebula, followed by re-accretion and (possibly) brecciation^32^,^33^). Features such as shock-induced mobilization of sulphides in (apparently) ‘unshocked’ OCs^32^, pyroxene polymorphs in CC matrices that require a high-temperature origin (>1,300 K) followed by very rapid cooling^33^,^34^ and matrix olivines showing dislocations indicative of significant shock deformation with adjacent chondrule olivines unaffected^9^,^35^, are entirely consistent with our observations. In addition, flattened, oriented or indented chondrules are a feature of many primitive meteorites, even those characterized as very weakly shocked (for example, Renazzo, S2; Fig. 5). In our modelling these chondrule textures become apparent at 2 km s^−1^ (Fig. 1b). Chondrule *P*(peak) in the forsterite simulation is 6.5 GPa (6.1 GPa with serpentine matrix): according to macroscopic shock indicators in chondrules, this is consistent with shock level S2 (very weakly shocked). CR chondrites are among the most pristine CCs: we might expect them to preserve the most complete record of early compaction. The detailed isotherm map generated from our TEM study of CR chondrite matrix revealed the degree of heterogeneity in GRA 06100. Although (as expected in a C2 chondrite) a large fraction of matrix shows little evidence of heating, mineral indicators in >50% of matrix are consistent with temperatures >900 K, and we are able to define areas that experienced *T*>1,180 K (ref. 24). We observe 1,000 K heterogeneity on 100 μm lengthscales. In a highly unequilibrated meteorite this required rapid heating and cooling. Chondrules show no evidence of shock metamorphic textures. These observations are all consistent with our model predictions. In the case of GRA 06100, they are a match to a ~1.5-km s^−1^ scenario with 70% initial matrix by volume. Finally, a specific model prediction—local melting of matrix in the most compacted primitive chondrites—has also been observed. Leoville is a reduced CV chondrite with porosity of 2% (ref. 14). A suite of observations, including highly variable shock effects on 100-μm scales, and local matrix melting, was explained by a combination of hot accretion and impact^36^. Our modelling suggests a simpler, unified explanation.

The apparent connection between shock level (increasing from CCs, through unequilibrated ordinary chondrites, to equilibrated ordinary chondrites (EOCs)) and asteroid metamorphism^1^,^2^ has prompted some to suggest that asteroid thermal metamorphism was a result of large impacts^37^. Mesoscale simulations showing PT variability with matrix fraction provide an alternative explanation. At an impact velocity of 2 km s^−1^, chondrule interiors in a CC-like (high matrix fraction) precursor experience *P*(peak) of ~5 GPa; an OC-like (low matrix fraction) precursor ~12 GPa; and a non-porous dunite (EOC approximation) ~24 GPa. In other words, shock level varies from S1–S4 depending on matrix fraction for the same impact velocity. Shock level is derived from observation of textures in chondrule olivines. There is a widely held view that varying shock level informs us about varying intensity of asteroidal collisions. But our data suggest that the way we use macroscopic textures to infer shock level can bias our interpretation. It is not required that varying impact energy caused the differences in shock level between the chondrite groups. Rather, varying matrix proportion in chondrite targets controlled the level of shock metamorphism recorded by the chondrules within them.

Both in terms of locating impact-induced compaction within the meteorite record, and exploring its significance with respect to the dynamical evolution of planetesimals, it is useful to constrain the timing of compaction. Chondrule formation defines an upper limit on chondrite accretion times. Chondrites could not have accreted prior to the formation of the youngest chondrules that they contain. In addition, it is apparent from CV^7^,^15^ and CR (this study) data that compaction occurred after aqueous alteration. Recent ^207^Pb–^206^Pb ages for CV calcium-aluminium inclusions (CAIs) (the assumed starting point for solar system formation) are 4567.18±0.50 Myr^38^ and 4567.30±0.16 Myr^39^. Using current ^238^U/^235^U values^38^ to correct published chondrule ages drives chondrule Pb-isotopic ages 0.95 Myr younger: 4,564.5±0.50 Myr for a multichondrule fraction from Allende^40^, 4,563.23±0.66 Myr for a multichondrule fraction from a CR^41^ (we can anticipate that individual chondrules within that fraction would have formed later than the 4,563.23±0.66 Myr average). Analysis of two individual Allende chondrules generated ages of 4,567.32±0.42 Myr and 4,566.24±0.63 Myr^39^. Relative ^26^Al/^27^Al chondrule ages agree, indicating that CC chondrules were still forming at 4.3 Myr after CAI^42^. Recent ^53^Mn–^53^Cr ages for secondary fayalite in CVs are 3.9(+1.0/−0.9) Myr^43^. Together, these data allow us to say that CC compaction did not occur earlier than 4–5 Myr after CAI. The OC data are more variable. Three chondrules from NWA 5697 have ages that range from 4,566.67±0.43 to 4,564.71±0.30 Myr^39^. ^26^Al/^27^Al suggests that the youngest OC chondrules date to ~3.5 Myr after CAI^42^. And ^53^Mn–^53^Cr for secondary fayalite from an OC gave 2.4(+1.8/−1.3) Myr^44^ after CAI. On balance, these data suggest an upper bound for compaction of OC planetesimals at ~3.5 Myr after CAI. Considering the chronometry data, our mesoscale simulations allow us to define a ‘speed limit’ constraint on the major compressive impact event(s) affecting CC planetesimals after 4–5 Myr, and OC planetesimals after ~3.5 Myr.

A spectrum of meteorite data (porosities, meteorite fabrics, petrography and local PT heterogeneity) are consistent with compaction from high *φ*_mi_ at velocities in the range 0.75–2 km s^−1^ (bulk *T*(final) ~400–700 K). In the case of CC-like simulations, velocities >2 km s^−1^ generate bulk *T*>700 K (evidence for metamorphism at these temperatures is extremely rare in CCs). In the case of OC-like simulations, velocities >1.25 km s^−1^ (corresponding to bulk *T*>600 K) generate matrix *T*(final) such that >20% of matrix is heated above the solidus (OC meteorites do not show evidence for widespread matrix melting). To translate our mesoscale observations into a constraint on asteroid-scale impact velocity, we can go to our macroscale numerical simulations of planetesimal collisions (Figs 3 and 4) which account for shock-wave attenuation with distance. Material in the range 400 K<bulk *T*(final)<700 K (blue) is consistent with meteorite data, according to the mesoscale simulation predictions. Material that experienced bulk *T*(final) >700 K (green and red) is not consistent with data from primitive unequilibrated meteorites. Of the material that is compacted to meteorite-like porosities, we assume that if >20% experienced high bulk *T*(final), and/or widespread matrix melting (for example, in OC targets), it would be observed in the meteorite record. In the example suite of simulations shown in Fig. 4 it is apparent that if asteroid collisional velocities exceeded 3 km s^−1^ in head-on impacts during the compaction phase (translating to ~4 km s^−1^ for average 45° impacts) this boundary would be crossed. Put another way, mesoscale simulations showing that velocities >2 km s^−1^ for CC impacts and >1.25 km s^−1^ for OC impacts are incompatible with meteorite data would translate to velocities of 3–4 km s^−1^ in real asteroid collisions.

Although the specific collisional ‘speed limit’ will depend on a variety of model assumptions, it is apparent (a) that initial impact velocities for OC and CC parent bodies were similar, and (b) that they must have been low. The chondrule age upper limit on accretion time, and chronometry for secondary olivine, indicates that these general constraints applied from ~4 Myr after solar system formation. In addition to relevance for understanding the early evolution of primitive solar system objects, our ‘speed limit’ has implications for dynamical models of giant planet migration. According to recent models^45^,^46^, OC-like parent bodies formed at 0.7–3 AU and CC-like bodies formed between the giant planets and from 8.0 to 13.0 AU. The migration of Jupiter resulted in an early (4–5 Myr) depletion and excitation of the asteroid belt region^45^,^46^. It was then repopulated with material from these two source regions^45^,^46^. Estimates for asteroid collisional velocities at 4 Myr from models that do not involve early migration of Jupiter^47^ are 5–9 km s^−1^. Grand Tack^45^,^46^ variants would produce higher mean impact velocities. This is not consistent with our observations. In addition, if OCs and CCs are derived from very different source regions we would expect to see evidence of rather different initial impact velocities. We do not. With respect to the planetary dynamics of the early solar system, our speed limit constraint is consistent with a relatively ‘late’ phase of main-belt collisional evolution, rather than the early, rapid depletion of a massive main belt^45^,^46^.

## Methods

### Numerical modelling

The iSALE shock physics code^17^,^18^,^19^ was used in two ways to quantify the compaction of porous planetesimal material in an impact. In one suite of models, 2D plane-strain, ‘mesoscale’ simulations were performed of shock wave propagation through a bimodal mixture of non-porous disks (chondrules) surrounded by a highly porous continuous matrix. The purpose of these simulations was to quantify the heterogeneity in peak pressure, post-shock temperature and post-shock porosity caused by the propagation of a single shock wave through a heterogeneous bimodal ‘chondritic’ mixture. In another suite of models, 2D axially symmetric, ‘macroscale’ simulations were performed of head-on collisions between porous planetesimals of different sizes and at speeds of a few km s^−1^. The purpose of these simulations was to constrain the post-impact distribution of compacted material consistent with meteoritic evidence.

### Mesoscale simulations

Chondritic meteorites are comprised of (nominally) zero-porosity spherical chondrules (0.1–1 mm in size) set in a highly porous matrix aggregate composed of sub-μm monomers. The three orders of magnitude difference in lengthscale of these two components allowed us to simulate shock in this material by explicitly resolving the chondrules as disks of non-porous forsterite in the 2D computational grid and surrounding these with a high-porosity continuum of forsterite (in 16 simulations), and serpentine (in 5). The porosity in this continuous matrix is parameterized as it exists on a lengthscale too small to be resolved in the simulation. The epsilon–alpha porous compaction model^19^,^48^ was used to parameterize the effect of pore space compaction during the shock, while an ANEOS equation of state table for dunite/forsterite^20^ was used to describe the thermodynamic response of the chondrule material. The non-porous part of the porous matrix material was described by either the dunite/forsterite equation of state, or an ANEOS equation of state table for serpentine^21^. A strength model for geologic materials^18^ was used to represent the response of the chondrules and matrix to changes in deviatoric stress: the chondrules were given a high cohesive strength (1 GPa), whereas the porous matrix was assumed to be very weak, with a cohesive strength of 100 kPa. A complete listing of material parameters used for the chondrules and matrix is given in Table 2. To generate a bimodal mixture analogous to the chondrule/matrix mixtures in chondritic meteorites, we randomly placed chondrule analogues (dunite disks) of various sizes ranging from 0.3 to 1 mm in diameter into an otherwise continuous region of the porous matrix analogue until the desired chondrule volume fraction was achieved. All simulations assumed an initial temperature of 300 K and an initial matrix porosity of 70%, apart from selected comparison runs at 60 and 80%. The remaining initial conditions are summarized in Table 2.

**Figure 6:**
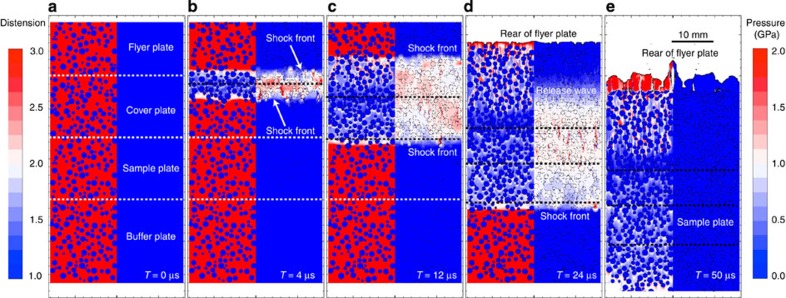
Time sequence of a typical mesoscale impact simulation. The nominally planar shock wave is shown propagating through a bimodal mixture of explicitly resolved non-porous chondrules surrounded by a high-porosity matrix, with snapshots showing the initial state (**a**), at 4 μs (**b**), 12 μs (**c**), 24 μs (**d**), and ending at 50 μs (**e**). Scale bar, 10 mm. Each time snapshot is divided into two panels, with colour-scales denoting distension (1/(1−porosity)) (high initially in matrix), and instantaneous longitudinal stress (0 initial throughout the mixture). Upon impact, shockwaves are generated at the flyer–sample interface and propagate both down into the sample and up into the flyer plate, compacting the matrix. In this example, after ~24 μs the shock wave in the sample has reached the sample/buffer plate interface; by this time the shock in the flyer plate has reflected off the rear of the flyer plate as a release wave that propagates back through the flyer, sample and buffer plates. By 50 μs the release wave has left the computational domain and the post-shock state of the sample may be recorded. The variation in peak pressure, peak- and post-shock temperature experienced by both the chondrules and matrix was recorded for subsequent analysis as was the reduction in porosity in the matrix.

In the mesoscale simulations, numerical planar impact experiments were performed in which a flyer plate impacted a target, comprising a sample sandwiched between a cover plate above and a buffer plate below (Fig. 6). The flyer, cover, sample and buffer plates were all comprised of the same bimodal mixture of non-porous chondrule disks, surrounded by a high-porosity matrix. The presence of a cover plate allowed the planar shock wave to achieve a steady form before passing through the sample and then the adjacent buffer plate. The simulation time extended until the sample was released from high pressure by a release wave from the rear of the flyer plate (see Fig. 6).

Figure 6 illustrates the propagation of the shock wave from a representative numerical simulation. Upon impact an upward moving shock wave is generated in the flyer plate and a downward shock wave is generated in the cover plate. As the shock propagates though the cover plate it evolves to a steady wave as the shock front thickness and rise time increase to constant values determined by the mesoscale structure of the material. For the particle size, bulk porosity and shock pressure range in our simulations; the shock front thickness was ~1–3 chondrule diameters, consistent with front thicknesses determined by mesoscale simulations of granular material compaction^49^. A consequence of the initial increase in shock front thickness is that shock compaction is greatest at the impact plane and decays with distance until a steady wave is achieved. This is evident from the gradient in porosity in the cover and flyer plates near the interface between them. The thickness of the cover plate (a few chondrule diameters) was chosen to ensure that the shock wave was steady when it entered the sample; no gradient in compaction exists within the sample. The mesoscale structure also creates resonant oscillations around the steady wave amplitude, which result in the important heterogeneous temperatures within the sample that are the focus of our study. Such oscillations have been observed in laboratory experiments of layered composites of ‘hard’ and ‘soft’ materials^50^ and observed and modelled in porous granular materials^51^ and porous rocks^52^. The magnitude and duration of the oscillations depends on the impedance mismatch between the components in the system^50^, which is very large for the chondrule/matrix system studied here. It is important to note that while the duration of the shock in our numerical simulations is long compared with typical laboratory experiments, it is short compared with shock durations in km-scale impacts on planetesimals (the shock duration is appropriate for cm-scale impactors). However, a systematic verification study that varied the shock duration showed no significant effect of shock duration on either the mean or the variance of the shock temperatures recorded in the simulations, provided the shock duration was longer than the time necessary to achieve a steady wave. Hence, extrapolation of results to longer shock durations is justified. To record changes in temperature, pressure and distension (porosity), passive tracer particles were initially placed one per cell and subsequently followed the particle path of that material. Both peak and instantaneous pressure and temperature were recorded by the tracers, as well as the instantaneous matrix distension (1/(1−porosity)). Bulk temperature, pressure and distension were computed as volume-weighted averages of all tracers in the shocked sample plate. Note that a consequence of the heterogeneity in shock propagation is that the peak shock pressure recorded by each tracer throughout the duration of shock wave passage can be substantially higher than the instantaneous bulk shock pressure at any time. For example, in the simulation shown in Fig. 1b the instantaneous bulk shock pressure in the shock wave was approximately 3±0.3 GPa and yet the mean peak shock pressure experienced by chondrule and matrix material in the sample was 6.5±1.8 and 8.4±2.6 GPa, respectively. Variations in peak and post-shock state were visualized using contour plots (for example, Fig. 1) and cross-sections through the sample at specific time intervals.

In post-processing, the statistical data from the tracer particles were analysed to obtain the mean and s.d. of each variable in both the chondrule and the matrix analogues (see Table 1). Values of post-shock temperature and porosity were recorded just after the release wave had passed through the sample mixture (for example, ~50 μs in Fig. 6). Repeat simulations with the same input parameters but different resolutions (cells across the largest chondrule) and different random distributions of chondrules were performed. In all cases, changes in the mean values were well within the s.d. of that value in a single simulation. To reflect the variability in mean values for simulations with different particle distributions, values in Table 1 are given to three significant figures only.

**Figure 7:**
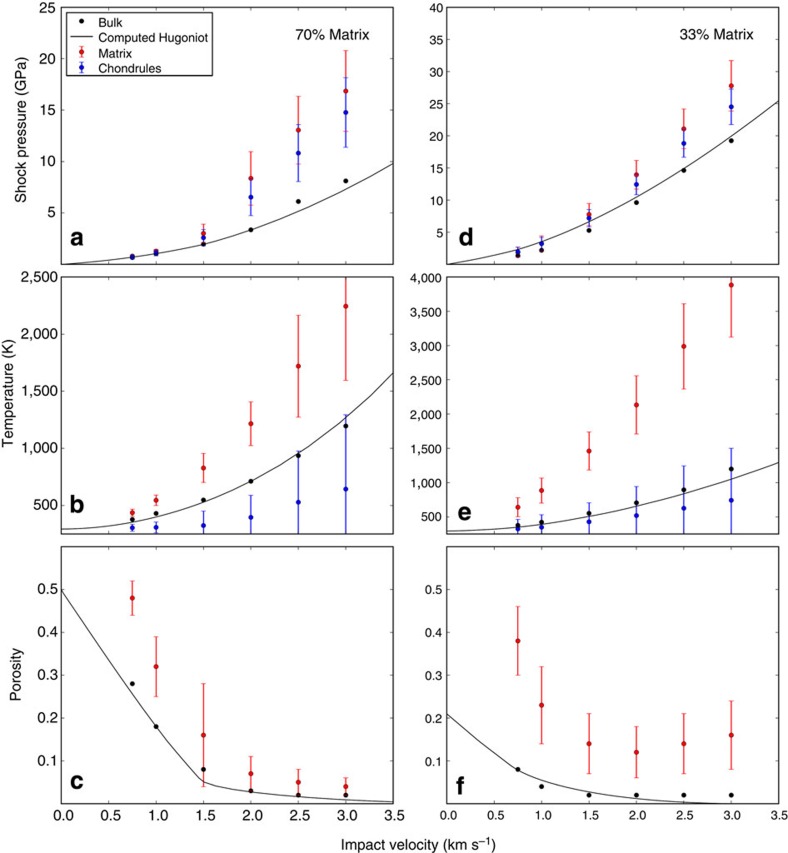
Pressure, temperature and porosity in mesoscale simulations. Shock pressure (**a**,**d**), post-shock temperature (**b**,**e**) and post-shock porosity (**c**,**f**) as a function of impact velocity for bimodal chondrule/matrix mixtures with matrix volume fraction 70% (**a**–**c**) and 33% (**d**–**f**). Black symbols show bulk values of the state quantity, averaged over the entire sample region; red and blue symbols (and error bars) show mean (and s.d.) of the state variable in the matrix and chondrule fraction, respectively. Apart from the peak-shock pressures in the matrix and chondrules, which represent the maximum pressure recorded by the material at any time, all variables are measured at a given instant in time either during the shock passage (bulk pressure) or after release (final temperature and porosity). As a result, while the bulk porosity and final temperature values represent volume-weighted averages of the porosity and temperature of the matrix and chondrules components, the bulk shock pressure values are substantially less than the peak-shock pressures recorded by matrix and chondrules. Solid line shows a Hugoniot curve for the bulk porous material (0.49 for 70% matrix; 0.23 for 33% matrix) computed using the epsilon–alpha porous compaction model.

Figure 7 illustrates the variation in the bulk shock response, as well as in the response of the matrix and chondrule components, with impact velocity. The peak shock pressure experienced by both the matrix and chondrules can be more than twice the bulk shock pressure of the steady shock wave. The massive difference in compressibility between the porous matrix and the non-porous chondrules results in large temperature differences between the matrix and chondrules. Impact velocities <4 km s^−1^ are not sufficient to compact all porosity from the matrix/chondrule mixture.

We note that the agreement between the macroscale parameterization of porous compaction (the computed Hugoniot; black solid lines in Fig. 7) and the bulk behaviour observed in the mesoscale simulations (black circles) demonstrates self-consistency between the mesoscale modelling approach and the bulk parameterization used in previous work^53^ and the macroscale models of impacts on a planetesimals (see macroscale modelling section). The difference in average peak shock pressure between the chondrules and matrix is a consequence of the large strength difference between the two components. While the average peak longitudinal stress is the same in the chondrule and matrix components, the much higher strength of the chondrules implies that the corresponding peak pressure (the isotropic part of the stress tensor) in the chondrule is lower than in the weaker matrix.

The mesoscale simulations described here provide a significant first step in quantifying the heterogeneous response of chondritic precursor material to shock compaction, which we will refine in future by addressing some of the simplifying assumptions used here. Principal among these is the use of 2D plane-strain geometry, rather than a more realistic three-dimensional (3D) geometry. This assumption was necessary to limit computational cost. In 3D geometry, out-of-plane contacts between chondrules would likely stiffen the bulk response of the mixture, particularly in scenarios where the initial chondrule volume fraction is high. However, based on similar numerical mesoscale studies of pore-space compaction in 2D and 3D^52^ we expect qualitatively similar behaviour in both geometries, particularly with regard to the magnitude and lengthscale of the PT heterogeneity and the trends in PT heterogeneity with both impact velocity and initial matrix fraction.

The porous compaction model and the dunite/forsterite and serpentine equation of state tables used to describe both the chondrules and solid-component of the porous matrix are also oversimplified. The compaction model assumes that all of the pressure–volume work deposited by the shock in the porous matrix leads to increase in temperature. In reality, dissipative processes during compaction, such as grain deformation and fracturing lead to an increase in entropy as well as temperature. Neglecting the entropy increase during crushing will result in an overestimate of shock heating, but this is difficult to quantify without experimental measurements of shock heating. The version of ANEOS used to derive the table does not permit both solid–solid and solid–liquid phase transitions to be included at the same time^54^. As in previous work^53^ the effect of the latter was regarded as less important than that of the former. Neglecting latent heat of melting implies that temperatures in the table that exceed the solidus are overestimated. In addition, as the real matrix is a multi-component system, the temperature increase in the matrix may be buffered by one of the less refractory components reaching the point of a phase change (for example, melting or vaporization) below the assumed melting point of our single-component chondritic analogue (1,373 K). As a first step to address this issue, we performed a complementary suite of mesoscale simulations that used a serpentine equation of state to represent the solid component of the matrix in place of forsterite (see Table 1). In low-velocity simulations there is little difference in matrix *T*(final) between serpentine and forsterite. In simulations ≥2 km s^−1^ temperature is buffered by phase changes in serpentine, reducing matrix *T*(final). However, still evident are high matrix temperatures alongside ‘cold’ chondrules, and significant PT heterogeneity over short lengthscales.

At higher shock pressures, shock compression experiments of quartz^55^ suggest that ANEOS overestimates the temperature increase and underestimates the entropy increase during shock compression, because it assumes a heat capacity in the fluid region that is too low^56^. If this limitation of ANEOS is also important for other silicate rocks, it implies that the shock pressure required to vaporize the matrix is overestimated by ANEOS and that peak- and post-shock temperatures above the liquids are also overestimated. As our primary focus here is relatively low-velocity collisions, which causes matrix heating below and up to the solidus, this limitation of ANEOS is of minor significance to our conclusions.

For the reasons described above, the peak- and post-shock temperatures quoted in Table 1, particularly those above the solidus, can be considered as upper limits for the given impact scenario. On the other hand, peak- and post-shock temperature are a strong function of initial temperature. A less conservative initial temperature in our models could easily compensate for any overestimate in temperature due to inadequacies of the material model. Moreover, the relative trends of increasing temperature with impact velocity and chondrule volume fraction are robust.

Of the assumed model parameters, peak- and post-shock matrix temperatures are most sensitive to the initial porosity of the matrix. Hence, the effect of initial matrix porosity on post-shock temperature provides a good measure of the sensitivity of the temperature estimates to all model assumptions. In simulations that varied the initial matrix porosity between 60 and 80%, we observed increasing post-shock matrix temperature with increasing initial porosity, for the same impact velocity (Table 1, main text). However, as demonstrated in Table 1, the mean post-shock matrix temperature for 60% initial matrix porosity is still 80–70% of the 80% initial matrix porosity values (at 1 km s^−1^ and 2 km s^−1^, respectively). Hence, temperature excursions are unlikely to be overestimated by >20–30% in our current simulations. Comparing the results of mesoscale simulations for the same 70% initial matrix porosity, but different impact velocities, a matrix temperature increase of 20–30% corresponds to an impact velocity increase of <1 km s^−1^. With regard to the ‘speed limit’ constraint provided by the lack of evidence for high matrix temperatures among meteorites, the uncertainty in matrix temperatures associated with a given mesoscale compaction scenario translates into an uncertainty in impact velocity of <1 km s^−1^.

### Macroscale simulations

In addition, a suite of ‘macroscale’ planetesimal collision simulations were performed. In macroscale simulations of planetesimal collisions, practical limits on computational mesh resolution did not allow us to explicitly resolve chondrule-scale heterogeneity in the planetesimals. Instead the material model of the chondrule–matrix mixture was approximated using the same approach as for the matrix material in the mesoscale simulations, but with strength and porosity parameters appropriate for the bulk material (see the ‘Bulk’ column of Table 2). Target planetesimals 200 and 500 km in diameter were modelled, with a uniform initial temperature (300 K). Two planetesimal bulk porosities were considered: 20%, which is appropriate for an ordinary chondrite (OC) parent body; and 50%, appropriate for a CC parent body. Impacting planetesimals of 10–150 km in diameter and with identical material properties to the target collided with the target at 1–10 km s^−1^.

In an impact on a real planetesimal, the shock wave attenuates rapidly with distance, but the peak-shock PT conditions are similar to the planar impact scenario. Tracer particles tracked planetesimal material exposed to different bulk post-shock temperatures (for example, Fig. 3) for comparison with the mesoscale simulation results and meteoritic constraints. The simplified material model used in our mesoscale simulations may overestimate the temperature excursions by as much as 20–30% (see supporting material), implying that an increase in impact velocity of up to 1 km s^−1^ (relative to those documented in Table 1) might be required to generate specific PT conditions.

### Electron microscopy

We studied the composition and mineralogy of matrix, opaque assemblages and chondrules. A JEOL JXA-8530 field emission gun ‘Hyperprobe’ was used to collect quantitative X-ray elemental maps and point analyses for Na, Mg, Al, Si, P, S, K, Ca, Fe, Ni and Co from a thin section using a 0.26-μm beam. Regions of matrix with distinct average chemical composition were identified. Broad-beam analyses of these regions were also obtained. These regions were also examined by FE-SEM to establish porosity, grain distribution, size and morphology.

TEM sections were prepared using a FEI Quanta 200 3D dual beam FIB workstation. FIB-TEM sections were examined using a JEOL 2010F field-emission gun TEM/scanning TEM equipped with an ultrathin window X-ray EDS. Four FIB sections were extracted from areas where the bulk composition and/or the porosity drastically changed. In addition, four other FIB sections from opaque assemblages were available from a previous study. Acquisition and processing of digital TEM images were conducted using GATAN’s Digital Micrograph imaging software. Quantification of EDS data was performed using the Cliff–Lorimer thin-film approximation. EDS spectra were collected in the scanning TEM configuration, using a 0.6-nm probe diameter. A counting time of 100 s was used to minimize the effects of beam damage and drift, while retaining meaningful counting statistics. After collecting each data point, each grain was examined using bright-field imaging. If the grain was not stable under the EDS probe for the 100-s duration of the analysis (that is, analysis resulted in a hole in the sample), the resulting analysis was not included in the data set. These analyses were excluded because it was unclear whether there was a beam overlap between the phase of interest and phase(s) lying underneath. Compositional heterogeneities were correlated with mineralogical markers, such as disequilibrium mineralogical assemblages, temperature-related crystallographic transitions, thermal decomposition of mineral phases and extent of solid solutions, to determine the scale of temperature heterogeneity.

FE-EPMA was carried out at the Regional Microanalytical and Imaging Center at Fayetteville State University. FIB and TEM analyses were conducted at the Material Characterization Laboratory at Pennsylvania State University.

## Author contributions

P.A.B. and G.S.C. originated the study and collaborated to explore potential implications, iterating towards a model appropriate for simulating chondrite compaction, and generating outputs that offered predictions against the chondrite record. P.A.B. led interpretation of the meteorite record and comparison with numerical results and wrote much of the paper. G.S.C. designed the mesoscale numerical approach described in this work. T.M.D. performed much of the numerical modelling and model analysis. N.M.A. performed combined microscopy and microanalytical study, producing the isotherm map of GRA 06100. F.J.C. contributed intellectual input on both numerical modelling and the meteorite record. A.R.M. and J.M. contributed to models involving transient heating, and detail on implications for palaeomagnetism.

## Additional information

**How to cite this article:** Bland, P. A. *et al.* Pressure–temperature evolution of primordial solar system solids during impact-induced compaction. *Nat. Commun.* 5:5451 doi: 10.1038/ncomms6451 (2014).

## Figures and Tables

**Table 1:** Results of mesoscale iSALE simulations of planar shock propagation through a chondrule/matrix mixture.

**Table 2:** Material parameters used in numerical simulations.

**Parameter**	**Chondrule**	**Matrix (forsterite)**	**Matrix (serpentine)**	**Bulk**
Initial porosity	0	0.6–0.8	0.6–0.7	0.5
Compaction rate*	NA	0.98	0.98	0.98
Vol. strain at onset of plastic compaction*	NA	−1E−5	−1E−5	−1E−5
Poisson ratio (solid component)†	0.23	0.23	0.23	0.23
Intact cohesion† (MPa)	1,000	0.1	0.1	10
Intact friction coefficient†	1.2	1.2	1.2	1.2
Intact strength limit† (GPa)	3.5	0.035	0.035	3.5
Damaged cohesion† (MPa)	0.01	0.01	0.01	0.01
Damaged friction coefficient†	0.6	0.6	0.6	0.6
Damaged strength limit† (GPa)	3.5	0.035	0.035	3.5
Melt temperature^3^ (zero pressure) (K)	1,373	1,373	1,098	1,373
Simon approximation constant‡ (GPa)	1.52	1.52	1.52	1.52
Simon approximation exponent‡	4.05	4.05	4.05	4.05
Thermal softening parameter†	1.2	1.2	1.2	1.2

NA, not applicable.

^*^Wunnemann *et al.*^19^

^†^Collins *et al.*^18^

^‡^Wünnemann *et al.*^57^
